# Glucocorticoid signaling mediates stress-induced migraine-like behaviors in a preclinical mouse model

**DOI:** 10.1177/03331024241277941

**Published:** 2024-08

**Authors:** Ya-Yu Hu, Rimenez Souza, Athithyaa Muthuraman, Leela Knapp, Christa McIntyre, Gregory Dussor

**Affiliations:** 1 Department of Neuroscience, School of Behavioral and Brain Sciences, The University of Texas at Dallas, Richardson, TX, USA; 2 The Center for Advanced Pain Studies, The University of Texas at Dallas, Richardson, TX, USA; 3 Texas Biomedical Device Center, The University of Texas at Dallas, Richardson, TX, USA; 4 Department of Biological Sciences, School of Natural Sciences and Mathematics, The University of Texas at Dallas, Richardson, TX, USA; 5 Department of Chemistry and Biochemistry, School of Natural Sciences and Mathematics, The University of Texas at Dallas, Richardson, TX, USA

**Keywords:** Restraint stress, glucocorticoids, corticosterone, glucocorticoid receptor, migraine, metyrapone, mifepristone, hypothalamic-pituitary-adrenal, mouse grimace score, von Frey test

## Abstract

**Background:**

Stress is one of the most common precipitating factors in migraine and is identified as a trigger in nearly 70% of patients. Responses to stress include release of glucocorticoids as an adaptive mechanism, but this may also contribute to migraine attacks. Here, we investigated the role of glucocorticoids on stress-induced migraine-like behaviors.

**Methods:**

We have shown previously that repeated stress in mice evokes migraine-like behavioral responses and priming to a nitric oxide donor. Metyrapone, mifepristone, and corticosterone (CORT) were used to investigate whether CORT contributes to the stress-induced effects. Facial mechanical hypersensitivity was evaluated by von Frey testing and grimace scoring assessed the presence of non-evoked pain. We also measured serum CORT levels in control, stress, and daily CORT injected groups of both male and female mice.

**Results:**

Metyrapone blocked stress-induced responses and priming in male and female mice. However, repeated CORT injections in the absence of stress only led to migraine-like behaviors in females. Both female and male mice showed similar patterns of serum CORT in response to stress or exogenous administration. Finally, administration of mifepristone, the glucocorticoid receptor antagonist, prior to each stress session blocked stress-induced behavioral responses in male and female mice.

**Conclusions:**

These findings demonstrate that while CORT synthesis and receptor activation is necessary for the behavioral responses triggered by repeated stress, it is only sufficient in females. Better understanding of how glucocorticoids contribute to migraine may lead to new therapeutic opportunities.

## Introduction

Among the various factors that trigger migraine attacks, stress is among the most common (1). Upon exposure to stressful conditions, individuals elicit a rapid and adaptive neurophysiological response aimed at preserving homeostasis through the activation of the hypothalamic-pituitary-adrenal (HPA) axis (2,3). Activation of the HPA axis initiates the release of corticotropin-releasing hormone (CRH) from the hypothalamus. This prompts the release of adrenocorticotropin releasing hormone (ACTH) from the pituitary, causing the secretion of glucocorticoids (GCs) from the adrenal gland (4). GCs regulate metabolic processes, cognitive functions, and behaviors that improve the chance of survival during homeostatic challenges (5). The process of adapting to acute stress, including the release of stress hormones to maintain homeostasis during changes, is known as allostasis (6,7). However, while this process can provide protective advantages in the short term, stress exposure over extended durations can lead to hormonal dysregulation (2,3,7,8). Allostatic overload due to prolonged exposure to stress or elevated glucocorticoid levels (6) can increase susceptibility to disorders such as anxiety, post-traumatic stress disorder (PTSD), and schizophrenia (9).

Prolonged or intense episodes of stress can cause abnormal hormonal fluctuations that lead to depression or anxiety (10). Many individuals with these disorders exhibit hyperactivity of the HPA axis, resulting in persistently elevated glucocorticoid levels (11). This sustained elevation impairs the negative feedback inhibition of glucocorticoids (12) and worsens the disruptive effects of these disorders (13,14). Besides the elevation of glucocorticoids, a precipitous decline in hormones can have disruptive effects on health and behavior. A rapid decline in corticosteroid levels is known to be problematic in drug therapy. Corticosteroids are widely used in the treatment of inflammation, autoimmune diseases, or chronic pain via activation of glucocorticoid receptors (GR) and mineralocorticoid receptors (MR) (15). The sudden withdrawal of corticosteroid administration following therapy can elicit adverse effects (16). Consequently, corticosteroid therapy is tapered off gradually. In contrast, many stressors end abruptly and may lead to rapid decline in corticosteroid levels, creating the very problem that slow tapering of drug dosing aims to avoid. Similarly, the decline in stress is significantly associated with migraine attack onset (17). Sudden termination of stress may lead to rapid decline in stress hormone levels and dysregulation of the fluctuation of glucocorticoids following repeated stress exposure may contribute to disease pathology. Ultimately, the role that stress-induced changes in glucocorticoid levels play in migraine remains unclear.

Better understanding of how stress contributes to migraine may lead to novel therapeutic approaches. Corticosterone (CORT) is the primary glucocorticoid responsible for regulating stress responses in rodents. The purpose of the studies described here was to investigate the role of CORT signaling on the migraine-like responses produced by repeated stress exposure in a mouse model. We hypothesized that fluctuations in CORT levels caused by repetitive stress might contribute to stress-induced migraine and that blocking these fluctuations could prevent stress-triggered migraine attacks. Using a preclinical stress-induced migraine model (18), we employed pharmacological tools such as metyrapone, an 11-beta hydroxylase inhibitor that blocks the conversion of deoxycorticosterone to corticosterone, and mifepristone, a glucocorticoid receptor antagonist commonly used in clinical and preclinical studies to assess the role of CORT synthesis and signaling in response to repeated stress.

## Methods

### Animals

All experiments were conducted with male and female outbred Institute of Cancer Research (CD-1) mice aged 6 to 8 weeks, acquired from Charles River Laboratories. They were housed in the vivarium at the University of Texas at Dallas, following a 12-hour light/dark cycle. To ensure consistency in weights within each experimental group, male and female mice were randomly assigned. Across all groups, male mice exhibited comparable average weights, as did female mice within their respective groups. Investigators were blinded to drug treatment groups in all experiments. All animal procedures were conducted following approval from the IACUC at The University of Texas at Dallas.

### Habituation and baseline testing

Each animal was handled for five minutes on the first three days. Subsequently, they were placed individually in 4 oz cups (Choice) inside the von Frey testing chamber and habituated for 2-hour per day over three consecutive days, as previously described (18). Beginning on the fourth day, animals underwent a 30-minute habituation period in the cups before baseline pain sensitivity testing commenced. Baseline pain sensitivity was assessed using von Frey filaments and facial expressions were evaluated using the mouse grimace score (as detailed in sections of Facial mechanical hypersensitivity and Spontaneous pain below).

### Restraint stress protocol

Stress exposure was performed according to methods described previously (18). Briefly, animals were placed in cylindrical tail access rodent restrainers (Stoelting 51338) and restrained for 2-hour per day for three consecutive days after baseline measurements were recorded. The restraint stress was carried out from 10:00 am to 12:00 pm each day, to not interfere with the natural rise in corticosterone levels in rodents after 1:00 pm (19). Once the animals were secured in the restraint tubes, they were checked every 30 minutes to ensure they had not shifted position or sustained any injuries. During the 2-hour period of restraint stress, the control animals were kept in their home cages without access to water or food. These control animals were housed in a separate room from the stressed animals throughout the duration of the stress protocol. After 2-hour of restraint stress, the stress group was returned to their home cages and food and water were provided to both groups. Stressed animals were not cohoused with control animals to avoid the transfer of a stressed phenotype between animals.

### Facial mechanical hypersensitivity

Mechanical thresholds were assessed via the application of manual von Frey filaments to the periorbital areas of the mice. Filament thresholds were determined using the 50% withdrawal thresholds calculated by the Dixon ‘up-down’ method (20). Testing began with 0.07 g. If no response was observed with the 0.07 g filament, the filament force was increased to 0.6 g. If a response was observed with the 0.07 g filament, the forces of filament were decreased until 0.008 g. Responses consisted of mice removing or swiping the filament away from the face when force was applied in the periorbital area. Animals that did not respond to the 0.6 g filament were at baseline. These animals were numbered and randomly allocated to different treatment groups.

### Spontaneous pain

Mouse grimace score (MGS) (21) was performed prior to von Frey testing for all time points to prevent potential influence of mechanical stimulation on the grimace response. Facial expression changes were based on five indicators, including orbital tightening, nose bulging, cheek bulging, flattening of ears and flattening of whiskers. These facial expressions were scored on a scale from 0 to 2 (0 = no grimace, 1 = somewhat grimace, 2 = maximal grimace). Each animal was recorded for three minutes by video camera (Logitech HD webcam C270) on each testing day. Experimenters were blinded to the treatments or groups when measuring grimace scores.

### Hyperalgesic priming

0.1 mg/kg sodium nitroprusside (SNP) (Sigma Aldrich, St Louis, MO) was prepared in 1X PBS solution. After returning to baseline following stress exposure as determined using the von Frey test, animals received an intraperitoneal injection of SNP or vehicle with a total volume of 150 μl. This component of the model tests for sensitization to nitric oxide, an important feature of a migraine model given donors of nitric oxide (NO) (18) reliably trigger migraine attacks in humans.

### Repeated corticosterone injections

Mice were randomly assigned to one of three experimental groups: 10 mg/kg of CORT, 20 mg/kg of CORT, or vehicle. Each mouse was administered subcutaneous injections of a total volume of 200 μl of CORT or vehicle for three consecutive days. The injections were performed at 10:00 am, followed by a 2-hour wait to simulate the duration of restraint stress. During this 2-hour period, food and water were removed in accordance with the restraint stress protocol. After the 2-hour period, animals were returned to their housing rooms. Additionally, apart from separating the group of mice receiving CORT from the vehicle group, mice receiving different doses of CORT were also housed in separate cages.

### Drugs

Each mouse received 50 mg/kg of metyrapone (Tocris Bioscience, USA) dissolved in 20% propylene glycol or vehicle with a total volume of 200 μl. For the repeated CORT injections experiment, CORT initially suspended in 2% dimethyl sulfoxide (DMSO) and diluted in peanut oil (Sigma Aldrich, St Louis, MO). Injections of 2% DMSO in peanut oil were used as a vehicle control. Animals were intraperitoneally administered mifepristone (10 mg/kg initially dissolved in 4% DMSO and diluted in corn oil, Tocris Bioscience, USA) or vehicle at a volume of 200 μl.

### Serum corticosterone measurement

The times of blood collection for the stressed animals were as follows: pre-stress (10:00), at 30 minutes into stress (10:30), at 2-hour into stress (12:00), 1-hour after the stress period (13:00), and 24-hour after the stress period (12:00 the next day) on the first and third stress days. Two protocols were used for the timeline of blood collection. In the first protocol, the animals experienced one day of restraint stress, and blood samples were collected at 10:00, 12:00, and 13:00 on the first stress day, and again at 12:00 the following day. In the second protocol, the animals underwent three consecutive days of restraint stress. Blood samples were collected at 10:00, 12:00, and 13:00 on the third stress day, and again at 12:00 the following day. At timepoints 30 minutes and 2-hour into stress (after the beginning of restraint), mice were removed from the restraint tube, euthanized, and blood samples were collected immediately.

For the CORT-injected animals, blood samples were collected at the following time points: vehicle (10:00), 30 minutes after CORT injection (10:30), 2-hour after CORT injection (12:00, during which the animals had no access to food or water to simulate stress conditions), 1-hour after the 2-hour period (13:00), and 24hour after the 2-hour period (12:00 the next day) on both the first and third injection days. We applied the same two protocols to the CORT injection groups as for the stress groups. In the first protocol, the animals received a single CORT injection. Blood samples were collected at 10:00, 12:00 and 13:00 on the first injection day, and again at 12:00 the following day. In the second protocol, the animals received CORT injections over three consecutive days. Blood samples were collected at 10:00, 12:00, and 13:00 on the third injection day, and again at 12:00 the following day.

To avoid adding to the stress response by taking blood repeatedly, we used separate groups of animals for each sampling point. Animals were anaesthetized by isoflurane and euthanized by decapitation. Trunk blood was collected and placed in microtubes. The microtubes containing blood samples were kept at room temperature for an hour for clot formation (22). Blood samples were centrifuged at 3000 × g for 10 minutes at room temperature, and the supernatant was collected as serum fraction. Serum CORT concentrations were measured using DetectX Corticosterone Enzyme Immunoassay Kit (Arbor Assays, MI, USA), following the protocol of the supplier.

### Statistical analysis

All data are presented as mean ± SEM. Behavioral data were analyzed for each time point using two-way repeated measures ANOVA followed by Bonferroni post hoc analysis. Serum CORT levels from the stress and CORT injection groups were analyzed using one-way ANOVA followed by Bonferroni post hoc analysis, while the mean CORT values for males and females at each time point were compared using unpaired t-tests. Data were analyzed using Prism 8 (GraphPad Software). Significance was set at P < 0.05 for all analyses. G Power was used to determine sample sizes in each experiment. Based on the previous experience with these models, an effect size of 1.5 was chosen, and the power was set at 0.8. It was determined that a minimum of eight mice per treatment group was required to observe statistically significant differences between groups. No mice were excluded after baseline exclusion. No data points were excluded in this study. For complete statistical analysis see Online Supplementary Table 1. All artwork was created using Biorender.

## Results

### Administration of metyrapone prior to each restraint stress session blocks behavioral responses in male and female mice

**Figure 1.**
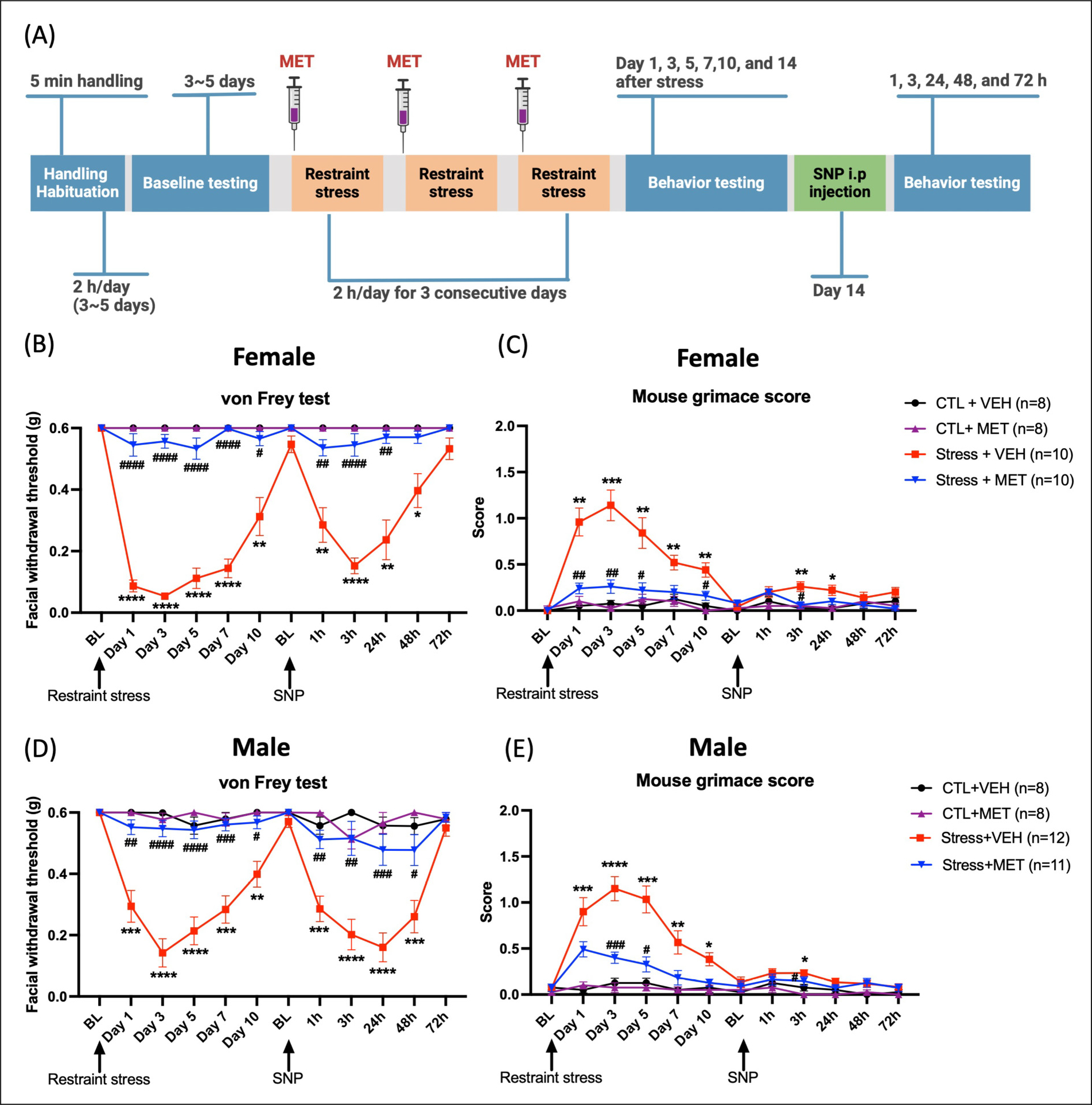
Administration of metyrapone blocks restraint stress-induced facial hypersensitivity in female and male mice. (A) Experimental timeline: Mice were injected with either 50 mg/kg metyrapone or vehicle 40 minutes prior to restraint stress. Upon returning to their baselines 14 days after stress, all mice were administered 0.1 mg/kg the NO donor SNP. Two-way ANOVA followed by Bonferroni multiple comparison analysis indicated significant differences between stressed and control mice in both males and females on both the von Frey test and grimace score. (B and D) Facial withdrawal thresholds during the acute phase (post-stress) and priming phase (post-SNP) were significantly different in stressed mice that received metyrapone versus stressed mice that received vehicle. (C and E) Grimace scores were also significantly different between stressed mice that received metyrapone versus stressed mice that received vehicle. Data are represented as mean ± SEM. * indicates significance between Stress + VEH and CTL + VEH, and # indicates significance between Stress + VEH and Stress + MET. *, #P < 0.05, **, ##P < 0.01, ***, ###P < 0.001, ****, ####P < 0.0001. Abbreviations: MET: metyrapone, VEH: vehicle, CTL: control, SNP: sodium nitroprusside, BL: baseline. See Online Supplementary Table 1 for a complete statistical analysis.

Our previous studies have shown that repetitive stress exposure evokes facial hypersensitivity and priming to a low dose of the NO donor sodium nitroprusside (SNP) in both female and male mice (18). To examine whether GCs are involved in these effects, we blocked production of CORT using metyrapone, a glucocorticoid synthesis inhibitor that inhibits the enzyme 11 beta-hydroxylase. The timeline for the experiments is shown in Figure 1A. Metyrapone or vehicle was subcutaneously injected into mice 40 minutes prior to each stress session. Behavioral responses were evaluated at the following timepoints: days 1, 3, 5, 7, 10 and 14 post-stress. Stressed mice did not return to baseline until 14 days post-stress, after which mice were intraperitoneally injected with 0.1 mg/kg SNP. Stressed female mice treated with metyrapone showed von Frey responses similar to those of control mice. Additionally, there were statistically significant differences between metyrapone-treated and vehicle-treated stressed mice at multiple days post-stress (Figure 1B). For the priming phase, only stressed mice treated with vehicle showed mechanical hypersensitivity starting 1 hour and lasting to 48 hours post-SNP injection. Similar experiments were conducted in male mice. As shown in Figure 1D, the effects of metyrapone were similar to those observed in females.

We further evaluated the spontaneous migraine-like behavioral responses using the mouse grimace scale. Stressed female mice showed significant grimacing on day 3, 7 and 10 post-stress (Figure 1C). Stressed male mice showed significant grimacing from day 1, 3, 5 and 7 after stress (Figure 1E). For those stressed groups that were pretreated with metyrapone, neither female nor male mice showed grimace responses at any time point post-stress or post SNP. These data demonstrate that in both males and females, the inhibition of glucocorticoid synthesis blocks repetitive restraint stress-induced facial hypersensitivity and priming to SNP. We also compared facial hypersensitivity between males and females and found a significant difference on day 1 post-stress in the vehicle-treated stress group, though this difference was not observed at other time points (data not shown). Overall, the pattern of facial hypersensitivity did not show substantial differences between sexes. Additionally, there was no significant difference in the mouse grimace scores between males and females.

### Exogenous CORT administration mimics the effects of restraint stress in female but not in male mice

**Figure 2.**
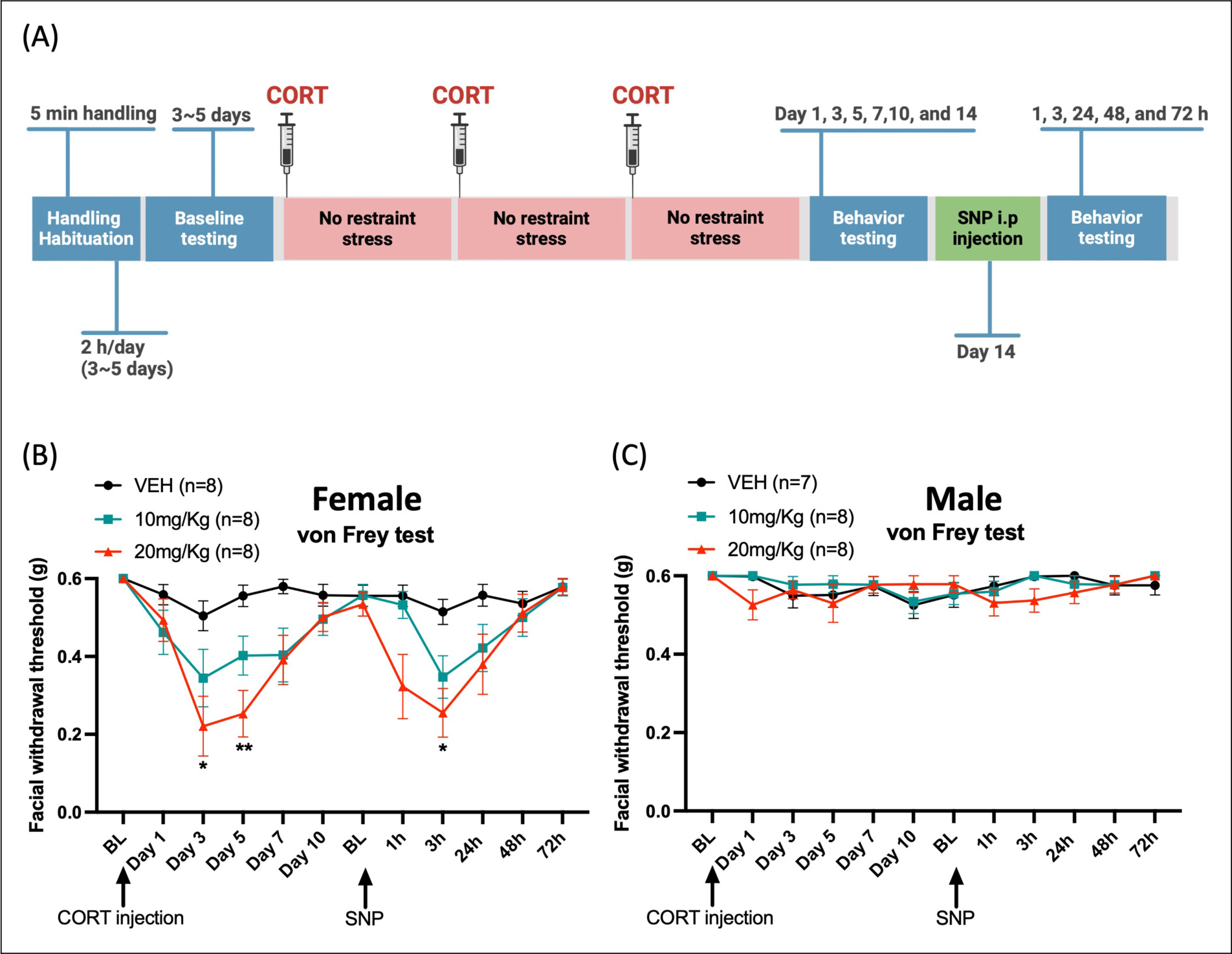
Repeated CORT injection induces facial mechanical hypersensitivity in female mice but not in male mice. (A) Experimental timeline: Mice were injected with either 10 mg/kg of CORT, 20 mg/kg of CORT, or vehicle for three consecutive days. Upon returning to their baselines 14 days after the last day of CORT injections, all mice were administered 0.1 mg/kg NO donor SNP. Facial withdrawal thresholds were measured in male and female mice after repeated CORT injections and priming with SNP. (B) Female mice showed a dose-dependent effect of CORT on facial withdrawal threshold. Two-way ANOVA revealed significant differences between 20 mg/kg CORT-injected female mice and vehicle injected mice and a Bonferroni post hoc analysis indicated differences during both the acute phase (post-injection) and priming phase (post-SNP injection). Although the group treated with 10 mg/kg CORT showed lower thresholds than vehicle-treated mice, the difference was not significant. (C) In male mice, there was no difference between the CORT-treated groups and the vehicle group. Data are represented as mean ± SEM. * indicates significance between CORT and VEH groups. *P < 0.05, **P < 0.01. Abbreviations: CORT: corticosterone, VEH: vehicle, SNP: sodium nitroprusside, BL: baseline. See Online Supplementary Table 1 for a complete statistical analysis.

To determine whether CORT is sufficient to induce the behavioral effects described above, we administered subcutaneous CORT injections for three consecutive days, as an alternative to the three-day restraint stress exposure. We tested 10 mg/kg and 20 mg/kg for these studies based on prior publications with these doses (23,24). Animals received either vehicle, 10 mg/kg or 20 mg/kg CORT through subcutaneous injections at 10:00 am on each of the three days (Figure 2A). As with the stress experiments, the animals were administered 0.1 mg/kg SNP on day 14 following the last CORT administration. In Figure 2B, female mice demonstrated a significant reduction in the facial mechanical withdrawal threshold in the 20 mg/kg CORT group on days 3, 5, and 7, as compared with the vehicle group. There were no significant changes in grimace scores at either 10 or 20 mg/kg CORT at any time point (data not shown), unlike that observed following repeated stress. Surprisingly, in males, 10 mg/kg and 20 mg/kg CORT failed to induce mechanical hypersensitivity responses or grimace after either CORT injections or after SNP injection (Figure 2C). Taken together, these findings demonstrate that CORT plays a potential role in the effects of stress in female mice, but other factors are responsible for stress responses in males.

### Influence of repetitive stress and daily CORT injections on serum CORT levels

**Figure 3.**
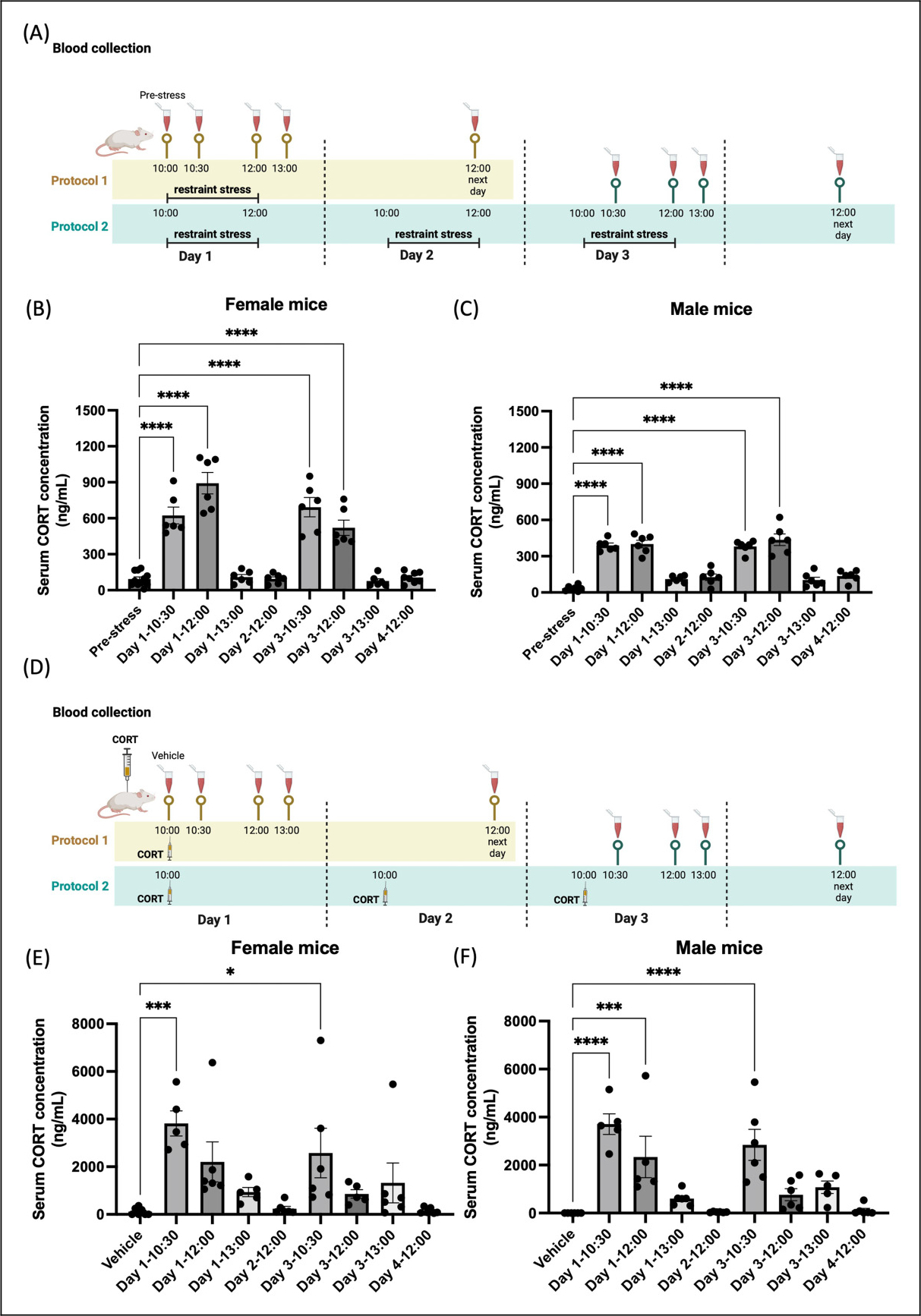
Serum CORT levels after repeated stress or CORT injections in male and female mice. (A) Experimental timeline: Mice were exposed to one day (protocol 1, yellow block) or three consecutive days (protocol 2, green block) of restraint stress. Blood samples were collected at time intervals indicated by sample tubes,10:00 (pre-stress), 10:30 (30-minute into stress), 12:00 (2-hour into stress), as well as 13:00 (1-hour after the stress period) and 12:00 the next day (24-hour after the stress period), on the first and third stress days. (B and C) On the first day of stress exposure, mice exhibited a significant increase in serum CORT levels at 10:30 (n = 6 females, n = 6 males) and 12:00 (n = 6 females, n = 6 males) after the onset of stress when compared to the levels at 10:00 (n = 11 females, n = 7 males). However, serum CORT levels returned to baseline at 13:00 (n = 6 females, n = 6 males). On the last day of the restraint stress, mice displayed a similar pattern to that observed on the first stress day. Serum CORT levels significantly increased at 10:30 (n = 6 females, n = 6 males) and 12:00 (n = 6 females, n = 6 males) timepoints following stress initiation, while CORT levels returned to baseline at 13:00 (n = 6 females, n = 6 males). (D) Experimental timeline: Mice were administered subcutaneous injections either as a single dose of CORT (protocol 1, yellow block) or over three consecutive days (protocol 2, green block.) Blood samples were collected at the same time intervals as the stress groups. (E and F) There was a significant increase in CORT levels at 10:30 (n = 5 females, n = 5 males) on day 1 CORT injection compared to the vehicle (10:00) baseline (n = 7 females, n = 6 males). CORT levels decreased at subsequent time points, including at 12:00 (n = 6 females, n = 5 males) and at 13:00 (n = 5 females, n = 6 males), ultimately returning to the baseline by the 12:00 next day (24-hour later) timepoint in both males (n = 6) and females (n = 6). On the final injection day, serum CORT levels mirrored those observed on the first injection day. Serum CORT levels significantly increased at 10:30 (n = 6 females, n = 6 males) on day 3 CORT injection and then began to decrease at 12:00 (n = 5 females, n = 6 males) and at 13:00 (n = 6 females, n = 5 males), ultimately returning to baseline levels at 12:00 next day (24-hour later) (n = 6 females, n = 6 males). One-way ANOVA followed by Bonferroni multiple comparison analyses were used. Data are represented as mean ± SEM. * indicate pre-stress vs. other time points in females and males in (B) and (C), and * indicate vehicle vs. other time points in females and males in (E) and (F). * P < 0.05, ** P < 0.01, *** P < 0.001, ****P < 0.0001. Abbreviations: CORT: corticosterone. See Online Supplementary Table 1 for a complete statistical analysis.

To evaluate whether CORT levels may be differentially regulated between the sexes, we measured serum CORT in stressed and CORT-injected mice. On the first stress day, female mice exhibited an increase in CORT levels at 30 minutes and 2 hours after stress (Figure 3A). Subsequently, the serum CORT level decreased and returned to the basal level 1 hour after the termination of stress, remaining stable for the following 24 hours. The last (third) stress day exhibited a similar trend, with female mice again demonstrating increased CORT levels at 30 minutes and 2 hours after stress, followed by a decline to basal levels at 1 hour and 24 hours after stress (Figure 3B). Male mice displayed a comparable pattern to female mice (Figure 3C). However, the overall magnitude of increase induced by stress was lower in male mice when compared to female mice only on day 1 at 2 hours following initiation of stress. These data demonstrate that stress exposure causes a transient increase in serum CORT levels, with a rapid decline, and a similar pattern between the sexes.

We next performed similar experiments to those described above but measured serum CORT following CORT injections (Figure 3D), which only caused behavioral responses in females. We observed a significant increase in serum CORT levels at 30 minutes after the first CORT injection in females. Subsequently, the serum CORT levels began to decline, returning to the basal level by 24 hours after the injection. This pattern of serum CORT was consistent with that observed on the third injection day (Figure 3E). In male mice, we observed a significant increase in serum CORT at 30 minutes and 2 hours post-injection, after which the levels decreased to baseline. On the third CORT injection day, we identified a comparable pattern of serum CORT level changes to the first CORT injection day (Figure 3F). Importantly, CORT injection led to levels of serum CORT that were much higher overall than stress-induced CORT levels (note the Y-axis on each graph). In contrast to stress, CORT injection led to peak serum CORT levels at 30 minutes while stress-induced peaks were later. Table 1 shows the mean ±SEM values of CORT levels in male and female mice, along with the p-values and the sample sizes for comparisons between the sexes at specific time points following the initiation of stress or CORT injection. Unpaired t-tests were conducted to compare the mean values between male and female mice. In the stress groups, significant sex differences were observed at the following time points: pre-stress, 10:30 and 12:00 on day 1, and 10:30 on day 3. In the CORT injection groups, no significant sex differences were detected at any of the time points following CORT administration. These data demonstrate that stress exposure and CORT injection leads to similar patterns of CORT elevation and decline in serum, with a more rapid decline and much higher overall levels following CORT injection. Additionally, stress leads to higher CORT levels in females than males at several time points.

### Administration of mifepristone prior to stress blocks stress-induced migraine-like behaviors in both male and female mice

**Figure 4.**
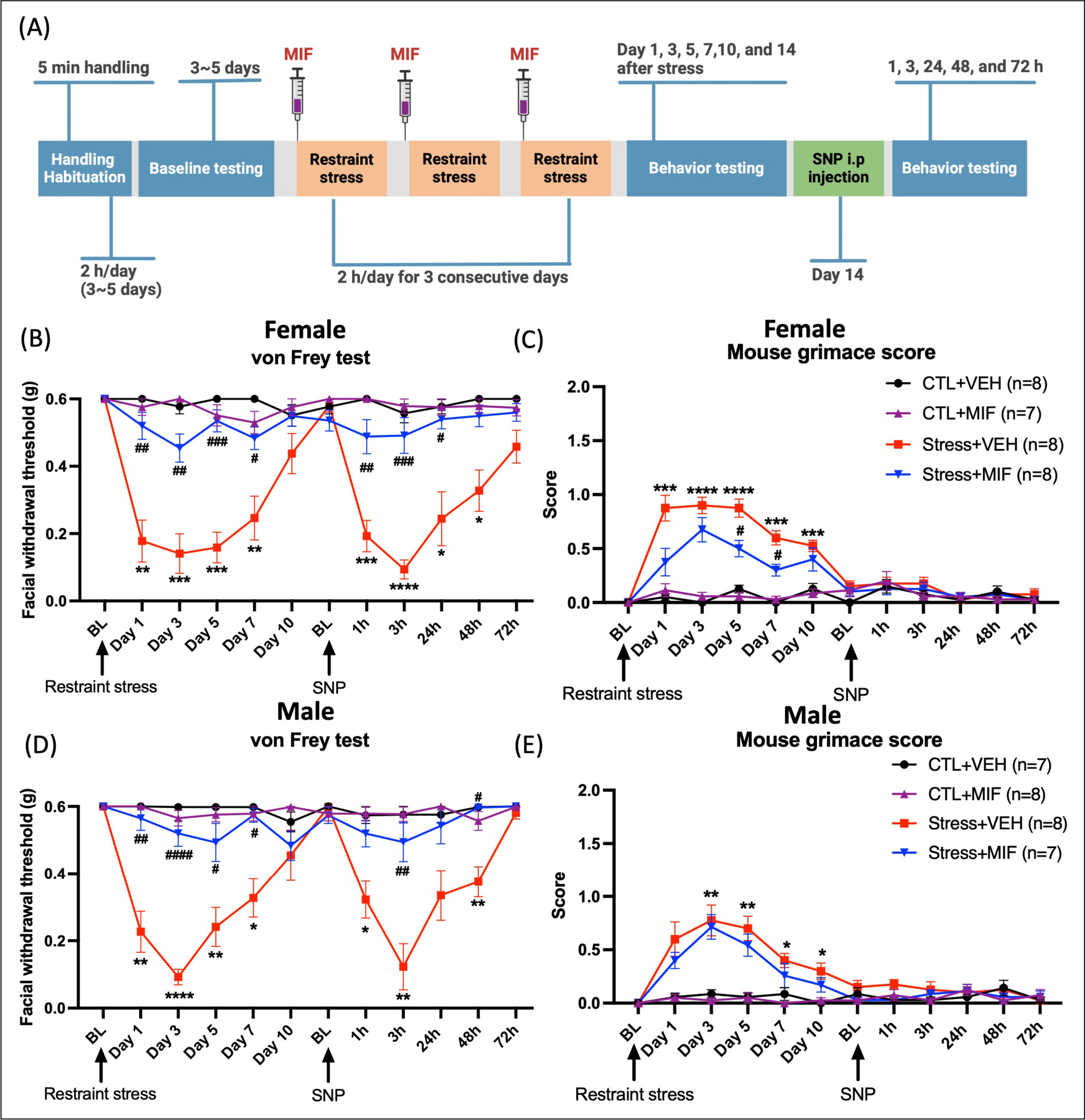
Pre-treatment with mifepristone before each stress session blocks stress-induced facial hypersensitivity in both male and female mice. (A) Experimental timeline: Mice were given intraperitoneal injections of mifepristone 40-minute prior to each stress session for 3 consecutive days. (B) Two-way ANOVA, followed by Bonferroni multiple comparison analysis, revealed a significant decrease in facial withdrawal threshold in stressed female mice pre-treated with vehicle vs. control mice pre-treated with vehicle during the acute phase (post-stress phase) and priming phase (post-SNP injection), while pre-treatment with mifepristone prevented facial hypersensitivity in stressed female mice. (C) The grimace data reveal a significant difference between the stressed mice pre-treated with a vehicle and the control mice pre-treated with a vehicle. Furthermore, a significant distinction was observed between the stressed mice pre-treated with a vehicle and the stress group pre-treated with mifepristone on day 5 and day 7 post-stress. (D) Male mice exposed to stress exhibited pronounced hypersensitivity both during the acute phase (post-stress) and the priming phase (post-SNP injection). In contrast, pre-treatment with mifepristone prevented stress-induced facial hypersensitivity in both phases. (E) Male mice exposed to stress exhibited significantly higher grimace scores than control mice. No significant difference was observed between the stressed mice treated with vehicle and the stressed mice treated with mifepristone. Data are represented as mean ± SEM. Statistical significance is indicated by asterisks as follows: *, #P < 0.05; **, ##P < 0.01; ***, ###P < 0.001; ****, ####P < 0.0001. Abbreviations: MIF: mifepristone, VEH: vehicle, CTL: control, SNP: sodium nitroprusside, and BL: baseline. See Online Supplementary Table 1 for a complete statistical analysis.

Since there were no major differences in the levels or patterns of serum CORT following stress or injection, we next asked whether sex differences in CORT responses may be mediated at the level of GRs. Mifepristone (RU486) is a commonly used inhibitor of GRs. Animals were administered 10 mg/kg of mifepristone 40 minutes before each stress session for three consecutive days (Figure 4A) (25). Female mice pre-treated with mifepristone and exposed to restraint stress showed significantly less hypersensitivity during both the post-stress and priming phases compared to vehicle (Figure 4B). Significant differences in grimace scores were observed between stressed mice pre-treated with vehicle and those pre-treated with mifepristone on days 5 and 7 post-stress (Figure 4C). Stressed male mice pretreated with mifepristone also showed significantly less hypersensitivity during the acute stress phase and priming phase (Figure 4D). However, there was no significant difference in grimace scores observed between the stressed mice treated with vehicle and those treated with mifepristone (Figure 4E). We further conducted comparisons between males and females and there were no significant differences in facial hypersensitivity or in mouse grimace scores between males and females at any of the time points examined (data not shown). These data demonstrate that stress acts, in large part, via the activation of GRs in both male and female mice.

## Discussion

**Figure 5.**
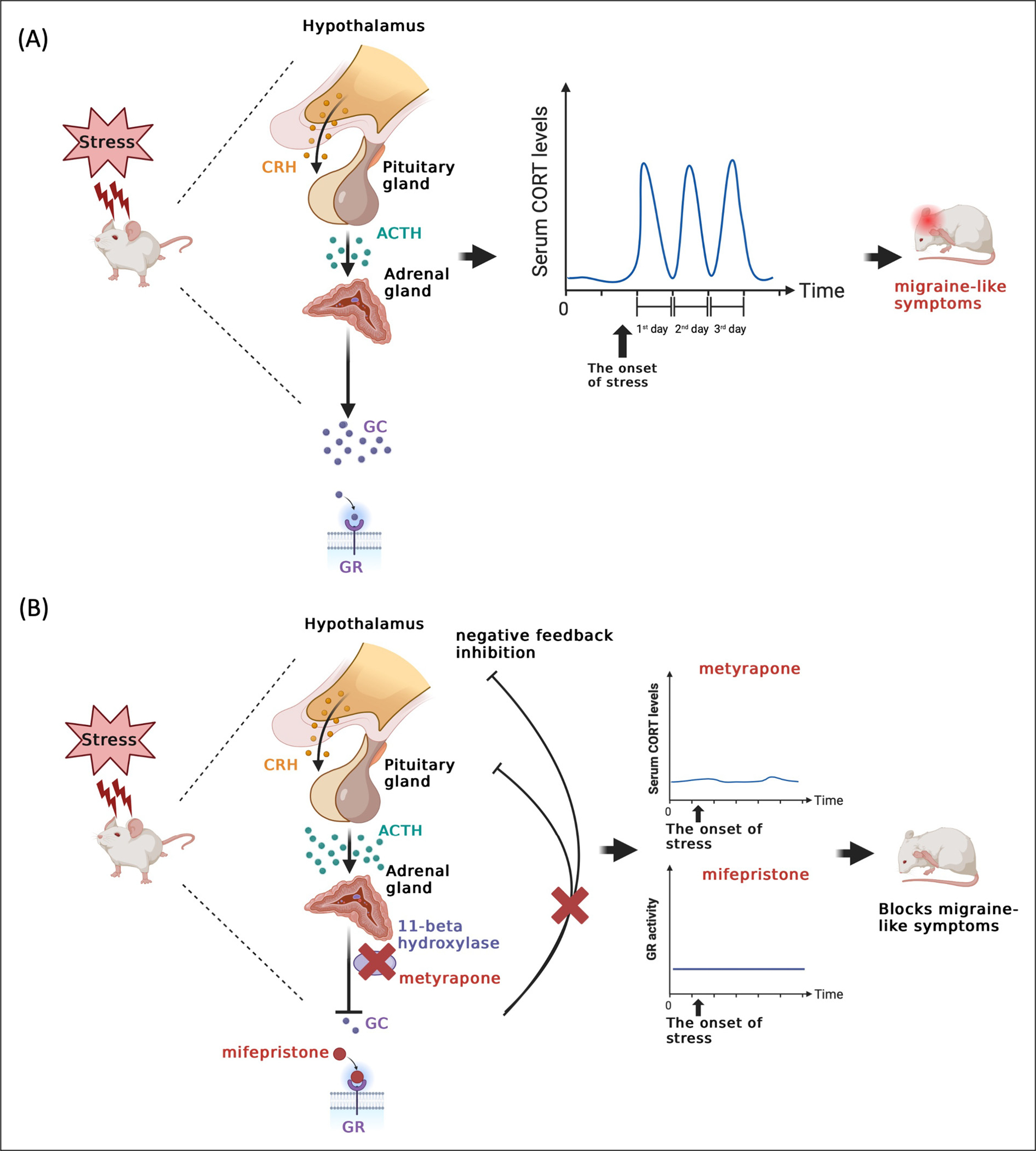
Fluctuations in glucocorticoid levels or receptor activity contribute to repetitive stress-induced migraine-like behavior in mice. (A) The scheme illustrates that stress triggers the activation of the HPA axis, subsequently leading to the release of CRH from the hypothalamus. CRH then binds to its receptor, initiating the secretion of ACTH from the pituitary gland. ACTH, in turn, targets its receptor, ultimately causing an increase in glucocorticoid levels from the adrenal gland. These glucocorticoids bind to their receptors, initiating downstream signaling pathways. Following the cessation of stress, glucocorticoid levels return to their basal level. Prolonged exposure to repetitive stress can result in fluctuations in glucocorticoid levels, potentially giving rise to maladaptive responses such as migraine attacks. (B) This schematic representation highlights that the administration of a glucocorticoid synthesis inhibitor, specifically metyrapone, which functions by inhibiting 11-beta hydroxylase or blocking the GR signaling pathway, can effectively suppress the fluctuation of glucocorticoid levels induced by stress. This inhibition has the potential to prevent stress-induced migraine-like behavior in a mouse model.

While the precise mechanisms linking stress to migraine attacks remain elusive, our findings using preclinical models establish that GCs play an important role in the initial hypersensitivity following stress (Figure 5A) and to the establishment of priming to a NO donor. This conclusion is supported by the observations that administering either metyrapone or mifepristone prior to stress exposure effectively blocked behavioral responses during both the acute stress and priming phases (Figure 5B). In females, exogenous CORT administration mimics the effects of stress but there was no effect of CORT injection in males. Thus, CORT synthesis is necessary for the behavioral responses to stress in both sexes, but it is only sufficient to induce a stress-like periorbital hypersensitivity response in females.

Stress and stress hormones have been found to directly impact glutamate neurotransmission, leading to an increase of neuronal excitability (26,27). Glutamate is thought to play a significant role in initiating and propagating cortical spreading depression (CSD), a phenomenon implicated in migraine mechanisms (28,29). Acute and chronic stress can increase susceptibility to CSD by reducing the threshold for its induction (30). Administration of CORT has been shown to exacerbate CSD susceptibility, particularly in Familial Hemiplegic Migraine type 1 (FHM1 R192Q) mice through activation of GR. However, acute restraint stress does not influence CSD frequency in R192Q or WT mice, despite elevating plasma CORT levels (31). This finding parallels our finding, indicating that the responses induced by CORT differ from those triggered by stress. In addition, stress may contribute to hypersensitivity within the peripheral nervous system (PNS). Numerous investigations have demonstrated that stress and GC can exacerbate pain resulting from nerve injury, with these effects being mitigated by GR inhibitors (32–34). One study revealed that exposure to stress or GCs prompts an increase in neurite growth within the dorsal root ganglion (DRG) via mechanisms involving GR-dependent gene transcription. Furthermore, CORT treatment has been shown to augment neurite length and complexity both in vitro and in vivo experiments (35).

The Table 1 revealed a significant sex difference in basal CORT levels in the stress groups at the pre-stress time point, with females exhibiting higher levels. This aligns with previous studies that have consistently shown females tend to have higher baseline plasma CORT levels (36,37). Furthermore, significant sex differences were observed at several time points in the stress groups. These findings indicate distinct differences in the stress response between males and females. Prior studies have shown distinct responses in the HPA axis between males and females (38–43). In the paraventricular nucleus (PVN), females exhibit greater numbers of Fos- and GR-positive cells than males after acute or repeated stress (44–46). Additionally, HPA axis-related gene expression is sexually dimorphic. Numerous studies have indicated that female rats express higher levels of CRH messanger ribonnucleic acid (mRNA) and proopiomelanocortin (POMC) mRNA, the precursor of ACTH, in the PVN, and in the central nucleus of amygdala in comparison to male rats after acute stress. Female rats also have higher plasma ACTH levels compared to male rats (45,47–49). Moreover, clinical studies have shown that chronic migraine (CM) patients exhibit irregular hypothalamic hormone secretion patterns, including elevated cortisol levels and a diminished nocturnal prolactin peak (50,51). Elevated levels of CRH in the cerebrospinal fluid of CM patients further support the involvement of the hypothalamus in chronic headache disorders (52). These studies demonstrate the presence and potential for sex-specific responses to stress within the nervous system, underscoring the importance of investigating how the hypothalamic system is involved in stress-induced migraine in the next step.

The explanation for the effect of CORT in female, but not male mice is not clear. Although the metabolism of exogenously administered CORT could differ between males and females, the absolute magnitude of serum CORT levels was much higher following CORT injection, compared to stress, in both male and female mice. Additionally, the return to baseline levels of CORT was more rapid in the stress condition but the decline in serum CORT was steeper following CORT administration. There was also a shorter lasting response to SNP on day 14 in the CORT-injected versus stressed females. Finally, CORT injections did not cause grimace responses. Thus, CORT injection does not fully reproduce the effects of stress even in females, suggesting that other factors are also involved, particularly in males. Repeated stress not only increases the level of GCs through HPA axis activation (5,53), but also engages multiple other physiological systems, including the autonomic nervous system, and the immune system (54,55), collectively contributing to the overall response (56).

It remains unclear whether stress itself or the termination of stress triggers migraine attacks, but stress is widely reported to be a major contributing factor to migraine attacks (1,57) and the accumulation of stress responses creates allostatic load that is proposed to contribute to migraine pathology (58,59). The response to stress is an immensely complex process and fully uncovering the mechanisms by which repeated stress exposure causes behavioral hypersensitivity requires more study. The present findings suggest that GR signaling plays a critical role and investigations into mechanistic links between glucocorticoids and migraine may lead to novel therapeutic approaches.

## Supplementary Material

Supplementary material

## Figures and Tables

**Table 1.** Illustrates a comparative analysis of average serum CORT levels across different time points for male and female mice exposed to stress or CORT injections.

The fluctuation in serum CORT levels prior to, during, and following exposure to stress.										

		Day 1 Stress				Day 3 Stress				
	Pre-stress	10:30	12:00	13:00	12:00 next day	10:30	12:00	13:00	12:00 next day	

Female	92.83±17.74	623.33±69.61	892.28±88.98	109.17±20.94	94.67±16.54	692.21±80.37	520.67±63.69	76.51±19.18	105.22±16.12	ng/ml
Male	34±8.16	389.73±19.08	400.83±31.51	110.58±11.31	126.44±26.78	380.88±20.69	436.15±47.77	103.26±22.88	135.93±19.15	ng/ml
*P value*	*0.0229* *	*0.0089* **	*0.0004* ***	*0.9537*	*0.3368*	*0.0038* **	*0.3134*	*0.3913*	*0.2414*	
n: F/M	11F/7M	6F/6M	6F/6M	6F/6M	6F/6M	6F/6M	6F/6M	6F/6M	8F/6M	

The fluctuation in serum CORT levels in the vehicle group or during and after the CORT injections.										

		Day 1 CORT injection				Day 3 CORT injection				
	Vehicle	10:30	12:00	13:00	12:00 next day	10:30	12:00	13:00	12:00 next day	

Female	115.31 ±56.89	3816.60±524.4	2203.67±841.0	937.16±191.8	236.45±98.85	2576.68±1042.0	858±181.6	1321.56±836.8	150.09±51.06	ng/ml
Male	0.64±0.1082	3708.60±429.4	2338.80±864.0	601.33±121.4	41.47±6.774	2845.17±645.8	766.42±249.3	1078.32±259.2	115.07±84.88	ng/ml
*P value*	*0.0907*	*0.8774*	*0.9138*	*0.1596*	*0.0774*	*0.8310*	*0.7816*	*0.8045*	*0.7310*	
n: F/M	7F/6M	5F/5M	6F/5M	5F/6M	6F/6M	6F/6M	5F/6M	6F/5M	6F/6M	

Data are presented as means±SEM. Comparison of mean values between males and females at various time points

* p<0.05

** p<0.0l

*** p<0.00l

Data are presented as means±SEM. No sex differences were observed in these groups.

## Data Availability

The data generated during and/or analyzed during the current study are available from the corresponding author on reasonable request.

## Key findings

- Preventing the synthesis of glucocorticoids by metyrapone blocks the long-lasting facial hypersensitivity observed after repeated restraint stress and priming to SNP.
- Repeated CORT injections over three consecutive days induce facial mechanical hypersensitivity exclusively in female mice, with no such effect observed in males.
- Both stress exposure and CORT injection induce comparable patterns of CORT elevation and decline in serum. However, CORT injection results in a more rapid decline and significantly higher overall levels compared to stress exposure.
- Blocking GR activation by administering mifepristone prior to stress exposure effectively blocks the development of facial hypersensitivity following stress and priming with SNP.
