# Supplementary material for: Glucocorticoid signaling mediates stress-induced migraine-like behaviors in a preclinical mouse model
Source: Cephalalgia. Author manuscript; Available in PMC 2024 Nov 20. (PMC11578425; doi:10.1177/03331024241277941)
Supplement: Supplementary material [file NIHMS2032266-supplement-Supplementary_material.docx]

**Supplementary table 1:** Statistical Analysis results

| Figure | Analysis | Statistics |
| --- | --- | --- |
| **Figure 1B**  von Frey test  Post-stress phase | Two-way repeated measure ANOVA  Bonferroni’s multiple comparisons between treatments | Interaction Factor: *F_(18, 192)_*=31.25; *p<0.0001*  Time Factor: *F_(3.096, 99.07)_*=36.44; *p<0.0001*  Treatment Factor: *F_(3, 32)_*=249.6; *p<0.0001* |
| **Figure 1B**  von Frey test  Post-SNP phase | Two-way repeated measure ANOVA  Bonferroni’s multiple comparisons between treatments | Interaction Factor: *F_(12, 128)_*=8.427; *p<0.0001*  Time Factor: *F_(2.814, 90.06)_*=10.90; *p<0.0001*  Treatment Factor: *F_(3, 32)_*=45.17; *p<0.0001* |
| **Figure 1C**  Grimace  Post-stress phase | Two-way repeated measure ANOVA  Bonferroni’s multiple comparisons between treatments | Interaction Factor: *F_(18, 192)_*=14.63; *p<0.0001*  Time Factor: *F_(2.423, 77.53)_*=28.46; *p<0.0001*  Treatment Factor: *F_(3, 32)_*=19.40; *p<0.0001* |
| **Figure 1C**  Grimace  Post-SNP phase | Two-way repeated measure ANOVA  Bonferroni’s multiple comparisons between treatments | Interaction Factor: *F_(12, 128)_*=1.514; *p=0.1273*  Time Factor: *F_(3.713, 118.8)_*=0.9040; *p=0.4583*  Treatment Factor: *F_(3, 32)_*=9.856; *p<0.0001* |
| **Figure 1D**  von Frey test  Post-stress phase | Two-way repeated measure ANOVA  Bonferroni’s multiple comparisons between treatments | Interaction Factor: *F_(18, 210)_*=14.79; *p<0.0001*  Time Factor: *F_(4.269, 149.4)_*=21.43; *p<0.0001*  Treatment Factor: *F_(3, 35)_*=40.08; *p<0.0001* |
| **Figure 1D**  von Frey test  Post-SNP phase | Two-way repeated measure ANOVA  Bonferroni’s multiple comparisons between treatments | Interaction Factor: *F_(12, 140)_*=7.132; *p<0.0001*  Time Factor: *F_(3.338, 116.8)_*=12.70; *p<0.0001*  Treatment Factor: *F_(3, 35)_*=21.28; *p<0.0001* |
| **Figure 1E**  Grimace  Post-stress phase | Two-way repeated measure ANOVA  Bonferroni’s multiple comparisons between treatments | Interaction Factor: *F_(18, 210)_*=11.45; *p<0.0001*  Time Factor: *F_(4.165, 145.8 )_*=26.24; *p<0.0001*  Treatment Factor: *F_(3, 35)_*=19.35; *p<0.0001* |
| **Figure 1E**  Grimace  Post-SNP phase | Two-way repeated measure ANOVA  Bonferroni’s multiple comparisons between treatments | Interaction Factor: *F_(12, 140)_*=0.7653; *p=0.6851*  Time Factor: *F_(3.012, 105.4)_*=5.905; *p=0.0009*  Treatment Factor: *F_(3, 35)_*=6.414; *p=0.0014* |
| **Figure 2B**  von Frey test  Post-CORT phase | Two-way repeated measure ANOVA  Bonferroni’s multiple comparisons between treatments | Interaction Factor: *F_(12, 126)_*=3.132; *p=0.0006*  Time Factor: *F_(4.329, 90.91)_*=15.24; *p<0.0001*  Treatment Factor: *F_(2, 21)_*=5.667; *p=0.0108* |
| **Figure 2B**  von Frey test  Post-SNP phase | Two-way repeated measure ANOVA  Bonferroni’s multiple comparisons between treatments | Interaction Factor: *F_(8, 84)_*=2.491; *p=0.0178*  Time Factor: *F_(3.347, 70.29)_*=9.395; *p<0.0001*  Treatment Factor: *F_(2, 21)_*=6.103; *p=0.0081* |
| **Figure 2C**  von Frey test  Post-CORT phase | Two-way repeated measure ANOVA  Bonferroni’s multiple comparisons between treatments | Interaction Factor: *F_(12, 120)_*=0.8885; *p=0.5606*  Time Factor: *F_(4.130, 82.60)_*=1.458; *p=0.2211*  Treatment Factor: *F_(2, 20)_*=0.3996; *p=0.6758* |
| **Figure 2C**  von Frey test  Post-SNP phase | Two-way repeated measure ANOVA  Bonferroni’s multiple comparisons between treatments | Interaction Factor: *F_(8, 80)_*=0.8759; *p=0.5405*  Time Factor: *F_(2.734, 54.69)_*=1.241; *p=0.3027*  Treatment Factor: *F_(2, 20)_*=1.713; *p=0.2057* |
| **Figure 3B**  Femlae Stress groups | One-way ANOVA  Bonferroni’s multiple comparisons between Pre-stress and other time points  Pre-Stress vs. D1: Stress 30 min  Pre-Stress vs. D1: Stress 2h  Pre-Stress vs. D1: 1h post-stress  Pre-Stress vs. D1: 24 post-stress  Pre-Stress vs. D3: Stress 30 min  Pre-Stress vs. D3: Stress 2h  Pre-Stress vs. D3: 1h post-stress  Pre-Stress vs. D3: 24h post-stress | *F_(8, 52)_*=45.43; *p<0.0001*  *****p<0.0001*  *****p<0.0001*  *ns, p >0.9999*  *ns, p>0.9999*  *****p<0.0001*  *****p<0.0001*  *ns, p>0.9999*  *ns, p>0.9999* |
| **Figure 3C**  Male  Stress groups | One-way ANOVA  Bonferroni’s multiple comparisons between Pre-stress and other time points  Pre-Stress vs. D1: Stress 30 min  Pre-Stress vs. D1: Stress 2h  Pre-Stress vs. D1: 1h post-stress  Pre-Stress vs. D1: 24 post-stress  Pre-Stress vs. D3: Stress 30 min  Pre-Stress vs. D3: Stress 2h  Pre-Stress vs. D3: 1h post-stress  Pre-Stress vs. D3: 24h post-stress | *F_(8, 46)_*=41.87; *p<0.0001*  *****p<0.0001*  *****p<0.0001*  *ns, p=0.2511*  *ns, p=0.0816*  *****p<0.0001*  *****p<0.0001*  *ns, p>0.9999*  *ns, p=0.0894* |
| **Figure 3E**  Female  CORT injected groups | One-way ANOVA  Bonferroni’s multiple comparisons between Pre-stress and other time points  Vehicle vs. D1: 30 min after injection  Vehicle vs. D1: 2h after injection  Vehicle vs. D1: 1h post-FWD  Vehicle vs. D1: 24 post-FWD  Vehicle vs. D3: 30 min after injection  Vehicle vs. D3: 2h after injection Vehicle vs. D3: 1h post-FWD  Vehicle vs. D3: 24 post-FWD | *F_(8, 43)_*=4.743; *p=0.0003*  ****p=0.0003*  *ns, p=0.0760*  *ns, p>0.9999*  *ns, p>0.9999*  **p=0.0207*  *ns, p>0.9999*  *ns, p=0.9932*  *ns, p>0.9999* |
| **Figure 3F**  Male  CORT injected groups | One-way ANOVA  Bonferroni’s multiple comparisons between Pre-stress and other time points  Vehicle vs. D1: 30 min after injection  Vehicle vs. D1: 2h after injection  Vehicle vs. D1: 1h post-FWD  Vehicle vs. D1: 24 post-FWD  Vehicle vs. D3: 30 min after injection  Vehicle vs. D3: 2h after injection Vehicle vs. D3: 1h post-FWD  Vehicle vs. D3: 24 post-FWD | *F_(8, 42)_*=12.19; *p<0.0001*  *****p<0.0001*  ****, p=0.0010*  *ns, p>0.9999*  *ns, p>0.9999*  *****p<0.0001*  *ns, p>0.9999*  *ns, p=0.4633*  *ns, p>0.9999* |
| **Figure 4B**  von Frey test  Post-stress phase | Two-way repeated measure ANOVA  Bonferroni’s multiple comparisons between treatments | Interaction Factor: *F_(18, 162)_*=9.925; *p<0.0001*  Time Factor: *F_(4.285, 115.7)_*=15.7; *p<0.0001*  Treatment Factor: *F_(3, 27)_*=48.50; *p<0.0001* |
| **Figure 4B**  von Frey test  Post-SNP phase | Two-way repeated measure ANOVA  Bonferroni’s multiple comparisons between treatments | Interaction Factor: *F_(12, 108)_*=4.139; *p<0.0001*  Time Factor: *F_(2.520, 68.04)_*=7.657; *p=0.0004*  Treatment Factor: *F_(3, 27)_*=41.45; *p<0.0001* |
| **Figure 4C**  Grimace  Post-stress phase | Two-way repeated measure ANOVA  Bonferroni’s multiple comparisons between treatments | Interaction Factor: *F_(18, 162)_*=9.922; *p<0.0001*  Time Factor: *F_(3.662, 98.87)_*=29.97; *p<0.0001*  Treatment Factor: *F_(3, 27)_*=53.52; *p<0.0001* |
| **Figure 4C**  Grimace  Post-SNP phase | Two-way repeated measure ANOVA  Bonferroni’s multiple comparisons between treatments | Interaction Factor: *F_(12, 108)_*=0.4857; *p=0.9193*  Time Factor: *F_(2.735, 73.84)_*=4.775; *p=0.0055*  Treatment Factor: *F_(3, 27)_*=0.7050; *p=0.5574* |
| **Figure 4D**  von Frey test  Post-stress phase | Two-way repeated measure ANOVA  Bonferroni’s multiple comparisons between treatments | Interaction Factor: *F_(18, 156)_*=9.211; *p<0.0001*  Time Factor: *F_(3.268, 84.98)_*=12.13; *p<0.0001*  Treatment Factor: *F_(3, 26)_*=77.29; *p<0.0001* |
| **Figure 4D**  von Frey test  Post-SNP phase | Two-way repeated measure ANOVA  Bonferroni’s multiple comparisons between treatments | Interaction Factor: *F_(12, 104)_*=5.840; *p<0.0001*  Time Factor: *F_(2.953, 76.79)_*=11.89; *p<0.0001*  Treatment Factor: *F_(3, 26)_*=23.11; *p<0.0001* |
| **Figure 4E**  Grimace  Post-stress phase | Two-way repeated measure ANOVA  Bonferroni’s multiple comparisons between treatments | Interaction Factor: *F_(18, 156)_*=5.126; *p<0.0001*  Time Factor: *F_(3.169, 82.38 )_*=18.13; *p<0.0001*  Treatment Factor: *F_(3, 26)_*=35.57; *p<0.0001* |
| **Figure 4E**  Grimace  Post-SNP phase | Two-way repeated measure ANOVA  Bonferroni’s multiple comparisons between treatments | Interaction Factor: *F_(12, 104)_*=1.022; *p=0.4345*  Time Factor: *F_(3.866, 100.5)_*=0.7468; *p=0.5582*  Treatment Factor: *F_(3, 26)_*=1.301; *p=0.2953* |
